# Developmental and hormonal regulation of Arabidopsis thaliana ornithine-delta-aminotransferase

**DOI:** 10.18699/VJGB-22-19

**Published:** 2022-03

**Authors:** A.A. Egorova, S.V. Gerasimova, A.V. Kochetov

**Affiliations:** Institute of Cytology and Genetics of the Siberian Branch of the Russian Academy of Sciences, Novosibirsk, Russia Novosibirsk State University, Novosibirsk, Russia; Institute of Cytology and Genetics of the Siberian Branch of the Russian Academy of Sciences, Novosibirsk, Russia Novosibirsk State University, Novosibirsk, Russia; Institute of Cytology and Genetics of the Siberian Branch of the Russian Academy of Sciences, Novosibirsk, Russia Novosibirsk State University, Novosibirsk, Russia

**Keywords:** ornithine aminotransferase, Arabidopsis thaliana, auxin, nitrogen, development, орнитинаминотрансфераза, Arabidopsis thaliana, ауксин, азот, развитие

## Abstract

Ornithine aminotransferase (OAT) catalyzes transfer of the delta-amino group from L-ornithine to oxo-glutarate. In plants, this reaction biochemically connects urea cycle, proline cycle, and polyamine biosynthesis pathway. OAT activity is shown to be associated with biotic and abiotic stress responses and nitrogen metabolism, but its physiological role is still unclear. In our study, we decided to investigate transcriptional regulation of the OAT gene in Arabidopsis thaliana under normal conditions and in response to various growth regulators. In the present work, the reporter gene construct containing the Escherichia coli β-glucuronidase gene (gus) under control of the A. thaliana OAT gene promoter was introduced into the genome of A. thaliana ecotype Columbia plants using the floral dip method; GUS activity was assayed in different experimental conditions including hormone treatment, low and high nitrogen and salinity. The GUS activity was analyzed histochemically. Plants were incubated with staining solution containing X-Gluc. We show that under standard growth conditions, the promoter is active during germination and in developing floral organs. OAT promoter activity specifically activates in response to different forms of auxin (IAA, NAA, and 2,4D), cytokinin (6- BAP), ethylene precursor (ACC), high nitrogen and salinity. Analysis of the OAT expression by qRT-PCR confirmed the pattern observed using the GUS reporter system. The OAT gene showed a significantly elevated expression in fourday- old seedlings and in plant roots in response to auxins and cytokinins. The analysis of the OAT promoter structure reveals cis-acting regulatory DNA elements associated with auxin regulation and abiotic stresses. The results of the study indicate that the OAT gene is involved in developmental processes and is regulated by auxin and cytokinins.

## Abstract (ru)

Фермент орнитинаминотрансфераза (OAT) катализирует перенос дельта-аминогруппы от L-орнитина на альфа-кетоглутарат. В растениях эта реакция связывает цикл мочевины, пролина и путь биосинтеза пролина. В литературе активность OAT связывают с ответом на биотические и абиотические стрессы, метабо- лизмом азота, но физиологическая роль этого фермента до сих пор остается неясной. В нашем исследовании мы изучали транскрипционную регуляцию гена OAT в Arabisopsis thaliana в нормальных условиях и в ответ на различные ростовые регуляторы. Репортерная конструкция, содержащая ген β-глюкуронидазы (gus) Escherichia coli под контролем промотора гена OAT из A. thaliana была интродуцирована в геном растений A. thaliana. Актив- ность GUS оценивалась в различных экспериментальных условиях, включающих воздействие гормонов, низких и высоких содержаний азота, солевой стресс. Для выявления активности GUS мы использовали гистохимический метод, растения обрабатывали раствором, содержащим X-Gluc. В нормальных условиях промотор был активен при прорастании семени и в развивающихся пестиках и пыльниках. Промотор гена OAT специфично активиру- ется в ответ на различные формы ауксина (IAA, NAA, 2,4D), цитокинина (6-BAP), предшественника этилена (ACC), высокие концентрации азота и NaCl. Результаты анализа экспрессии гена ОАТ соответствуют наблюдаемому пат- терну активности промотора, полученному с использованием репортерной системы GUS. Экспрессия гена OAT значимо повышалась в четырехдневных проростках и в ответ на ауксины и цитокинины. При анализе структуры промотора гена OAT обнаружены цис-элементы, связанные с ответами на ауксин и абиотические стрессы. Наши результаты позволяют сделать вывод о связи гена ОАТ с процессами развития растений и о его регуляции аукси- нами и цитокининами.

## Introduction

The ornithine-δ-aminotransferase (OAT) is a mitochondrial pyridoxal-5-phosphate (PLP)-dependent enzyme that transfers an amino group from ornithine to oxo-glutarate with formation of glutamate-1-semialdehyde (GSA) and glutamate (Gerasimova et al., 2011b). Although the biochemical function of OAT is known, its biological role in plants is not fully understood. On the one hand, OAT is involved in metabolism of ornithine, which takes part in numerous biochemical processes in plants, such as arginine metabolism, synthesis of polyamines and alkaloids (Funck et al., 2008; Majumdar et al., 2015). On the other hand, one of the products of the reaction mediated by OAT, namely GSA, is involved in proline production. It readily interconverts into the cyclic 1-pyrroline-5-carboxylate (P5C), an intermediate in the proline biosynthesis, in a non-enzymatic fashion (Ginguay et al., 2017). Proline is involved in plant stress response (Kochetov et al., 2004; Hayat et al., 2012) and development (Kavi Kishor et al., 2015). It has already been shown in various experiments on several plant species that overexpression of the OAT gene is associated with increased proline content and resistance to abiotic stresses (Roosens et al., 1998, 2002; Wu et al., 2003). It is tempting to assume that OAT might link biological processes related to proline, ornithine and P5C metabolism, such as nitrogen recycling, stress response, secondary metabolism, growth and development.

We have previously shown that OAT overexpression in tobacco increases salt stress resistance. Interestingly, the level of proline accumulation in OAT overexpressing lines did not differ from that of WT plants under both normal and stress conditions, suggesting that OAT might contribute to stress resistance through processes not related to proline synthesis (Gerasimova et al., 2010). On a model of transgenic tobacco plants expressing GUS under the control of putative Arabidopsis thaliana OAT promoter we showed that the promoter activity is associated with meristems and zones of active growth (Gerasimova et al., 2011a). This observation suggests that the OAT gene might be involved in developmental processes. The present study aims to investigate transcriptional regulation of the OAT gene in A. thaliana under normal conditions and in response to various growth regulators.

## Materials and methods

Development of transgenic Arabidopsis harboring AtOAT promoter construct. The 1844 bp region upstream of the OAT gene translation start (TAIR, AT5G46180) was cloned in the promoterless vector pBI101 with the formation of the P1844 construct (Gerasimova et al., 2011a). The resulting vector contains the expression cassette harboring the β-glucuronidase (gus) reporter gene under the control of putative A. thaliana OAT promoter. A. thaliana plants ecotype Columbia were grown at 22 °C in a long-day growth conditions (16 h of light and 8 h of dark). Construct P1844 was transformed into Agrobacterium tumefaciens strain AGL0, which was used to transform A. thaliana by floral dip method (Clough, Bent, 1998). T1 transformants were screened on 1/2 MS agar plates containing 50 mg/L kanamycin, transferred to pots and grown to maturity until the T2 generation seeds were harvested. T2 seeds were germinated on 1/2 MS agar plates containing 50 mg/L kanamycin and resistant plants were tested for the presence of GUS activity by histochemical assay. Six independent transgenic T2 lines showing the presence of GUS activity in seedlings were selected for further experiments. Plants from the selected lines were grown to maturity and T3 generation seeds were harvested. Thus, six independent T3 transgenic lines have been obtained.

GUS staining. The histochemical staining method (Jefferson et al., 1987) was used to visualize GUS (Escherichia coli β-glucuronidase) activity in seedlings grown on agar plates and plant parts grown in soil (5-week-old plants). Whole seedlings and different plant parts were incubated in X-Gluc solution (2 mM X-Gluc, 50 mM NaPO4, pH 7, 0.5 % (v/v) Triton-X) for 24 h at 37 °C. Chlorophyll was removed by repeated washing in 70 % (v/v) ethanol. GUS activity was observed using a ZEISS Stemi 2000-C microscope coupled with an AxioCam HRc camera

Experimental treatments. Surface-sterilized seeds of six independent transgenic A. thaliana lines (T3) were germinated on MS plates supplemented with 1 % sucrose, 0.7 % agar. To detect promoter activity during germination, histochemical assay was performed for seedlings at 3rd, 5th, 6th and 14th day after sowing (DAS) on plates. For experimental treatments, one-week-old seedlings were transferred to the same medium supplemented with the following growth regulators (from Sigma-Aldrich): auxins (1 mg/L NAA, 2 mg/L IAA, 0.5 mg/L 2,4-D), cytokinins (1 mg/L 6-BAP, 100 μМ trans-zeatin, 10 μМ and 100 μМ kinetin), gibberellic acid (10 μМ GA3), 100 μМ abscisic acid, 1 mМ methyl jasmonate, ethylene precursor (50 μМ ACC), high nitrogen (10 mM NH4NO3), high salinity (200 mM NaCl). For low nitrogen treatment, MS NH4NO3-free medium (Duchefa Biochemie) was used. GUS activity was assayed after 1, 4, 6 and 8 days of treatment. For cold and heat treatment, two-week-old transgenic plants were used. For cold treatment, plates with seedlings were incubated at +4 °C for 4 h, then for 2 h at 22 °C; for heat treatment, plates were incubated at +50 °C for 15 min, then 6 h at 22 °C.

Gene expression analysis (RNA isolation and qRT‑PCR). Wild type Col-0 seed was surface sterilized with 12.5 % bleach (Aqualon) and 70 % ethanol and germinated on 1/2 MS medium (16-h daylight, 22 °C). To measure expression of the OAT gene during germination and early development, total RNA was isolated from whole seedlings at 4th, 7th and 14th DAS. For experimental treatments, one-week seedlings were transferred to 1/2 MS medium supplemented with different growth regulators (1 mg/L NAA, 2 mg/L IAA, 0.5 mg/L 2,4-D, 1 mg/L 6-BAP, 50 μМ ACC), and control, to 1/2 MS medium. For each treatment, experiment was performed in three biological replicates. There were 30 seedlings per each biological replicate. Total RNA was isolated from roots of seedlings after 6 days of treatment with the RNeasy Plant Mini Kit (Qiagen). RNA was treated with DNAse (QIAGEN RNase-Free DNase Set). The concentration of RNA was measured by NanoDrop 2000 (Thermo Scientific). The quality of RNA was evaluated using Bioanalyzer 2100 (Agilent). First strand cDNA was synthesized from 1 μg of total RNA using BIORAD iScript™ Reverse Transcription Supermix for RT-qPCR. For qRT-PCR analysis, cDNA was diluted ten times. PCR was performed in a final volume of 15 μL: 3 μL of 5х Low Rox buffer (SibEnzyme), 0.15 μL of each primer (10 μM) and the taqman probe solution, 3 μL of diluted cDNA. The primers and probes were designed using IDT’s PrimerQuest Tool (https://eu.idtdna.com/PrimerQuest/). The comparative threshold cycle method was used to determine relative gene expression, with the expression of EF1-alfa and F-box (accession no. At1g13320 and At5g15710) serving as an internal control. The structures of primers and probes are given in Suppl. Table 11. The relative expression levels of OAT mRNA in all the treated samples were quantified using an Applied biosystems 7500 Real Time PCR System. Each reaction was performed in three technical replicates using the following program of the qRT-PCR; 95 °C for 10 min; 45 cycles of 95 °C for 15 s, 68 °C for 60 s. Statistical analysis was performed using Student’s t-test. p-values < 0.05 were considered significant.

Supplementary Tables 1–4 are available in the online version of the paper: http://www.bionet.nsc.ru/vogis/download/pict-2022-26/appx4.pdf


Web tools used for cis-acting regulatory DNA elements search and expression data analysis. Search of cis-acting regulatory elements was performed using the PLACE database (Higo et al., 1999). Gene expression data from different microarray and RNA-seq experiments were extracted from Expression Atlas and Arabidopsis eFP Browser Web tools (Winter D. et al., 2007; Papatheodorou et al., 2018).

## Results

**Fig. 1.**
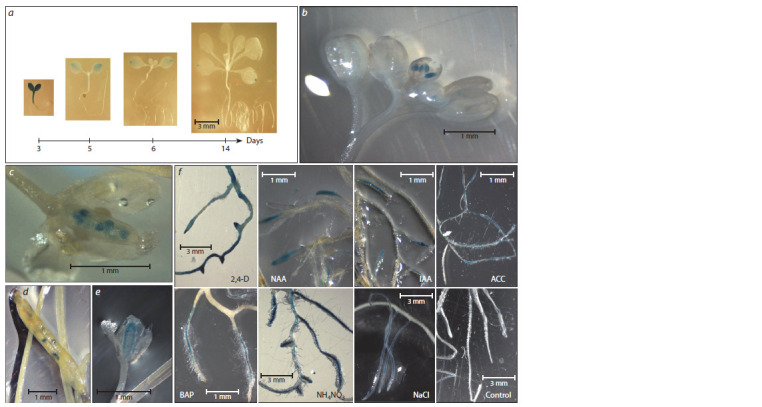
Histochemical GUS analysis of transgenic A. thaliana lines: a–e, tissue-specific GUS expression during plant development: a, different stages of seedling development; b, GUS expression in immature anthers; c, GUS expression in ovules; d, developing silique with GUS expression in seeds; e, developing carpel; f, roots of plants treated with different inducers. Age of plants on pictures b–e – 1.5 months, on picture f – 13 days.

Tissue-specific promoter activation at different developmental stages and under experimental treatments. Strong GUS staining was detected in hypocotyls and cotyledons of seedlings at 3–4th DAS. At later stages, the GUS activity was observed only in cotyledons. In 6- and 14- DAS seedlings, the GUS activity was found only in the distal parts of cotyledons (Fig. 1). During flower development, the GUS activity was observed in anthers, carpels and developing seeds of growing siliques (see Fig. 1, Suppl. Table 2).

To get a deeper insight into the transcriptional regulation of OAT, transgenic seedlings were subjected to experimental treatments including different concentrations of growth regulators and phytohormones auxins, cytokinins, gibberellin, ABA, methyl jasmonate, ethylene precursor (ACC), low and high nitrogen, high salinity, cold and heat stress (see Fig. 1, Suppl Table 2). We observed tissue-specific OAT promoter activity in response to different forms of auxin (IAA, NAA, and 2,4-D), cytokinin (6-BAP), and ACC. The strongest GUS activity was observed in response to 2,4-D in whole plant. Treatment with IAA and NAA caused GUS activation in root tips; treatment with 6-BAP – in the zone of root hairs. Treatment with ACC, salinity and nitrogen activated promoter along the whole root.

**Fig. 2.**
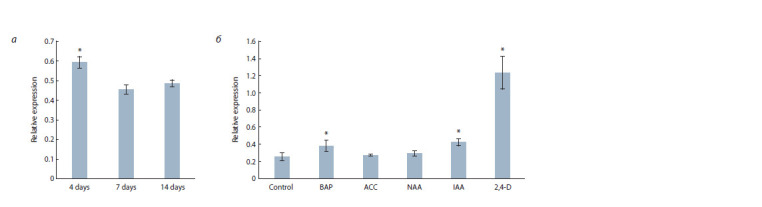
The relative expression of OAT: a, in whole 4-, 7- and 14-days seedlings; b, in roots of seedlings, which were grown for 6 days on medium with inducers. An asterisk indicates statistical signif icance in a one-way ANOVA ( p ≤ 0.05). Each bar represents the mean of three biological replicates ± SE.

OAT gene expression analysis. The qRT-PCR results showed that transcript levels of the OAT gene are significantly higher in four-day-old seedlings, than at later developmental stages (Fig. 2, a). In experimental treatments, the OAT gene showed significantly ( p ≤ 0.05) elevated expression in response to different forms of auxin (IAA and 2,4-D) and cytokinin (6-BAP) in comparison to control conditions. Treatment with synthetic auxin 2,4-D led to 3-fold increase in OAT expression level in roots (see Fig. 2, b).

Cis-acting regulatory DNA elements search and transcriptomic data analysis. Сis-acting regulatory DNA elements search revealed putative transcription factors binding sites, corresponding to different physiological processes, including hyperosmotic and hypoosmotic stress response, auxin response, axillary bud dormancy control, specific regulation in ontogenesis (Suppl. Table 3). Meta-analysis of microarray and RNA-seq data (Winter D. et al., 2007; Papatheodorou et al., 2018) shows that the OAT expression level changes in response to cold, drought, heat, wounding, osmotic and salt stress. Expression increases in response to pathogens Botrytis cinerea, Pseudomonas syringae, Phytophthora infestans and some other infections. Altered OAT gene expression was observed in response to different hormones: it increased in response to 3 h of treatment with ABA, methyl jasmonate, and decreased in response to 3 h of treatment with brassinosteroids. The OAT gene demonstrates high expression in seeds, siliques, embryos, senescent leaves, floral organs in A. thaliana (Suppl. Table 4).

## Discussion

For more than a decade OAT has been considered an enzyme involved in metabolic response to different stress conditions, such as osmotic stress, pathogen attack and ROS production, nitrogen starvation, etc. (Funck et al., 2008; Verslues, Sharma, 2010; Qamar et al., 2015). This enzyme belongs to the network of nitrogen-metabolizing pathways in plants, affected by various environmental stimuli. It has been shown that plants accumulate proline during stress conditions (Verbruggen, Hermans, 2008). The results of Funck et al. (2008) and our previous study did not support the hypothesis of OAT contribution to proline accumulation. Instead, a specific role of the OAT gene in plant developmental and growth processes under both normal and stress conditions is hypothesized (Gerasimova et al., 2010, 2011a). This study provides a deeper insight into the role of the OAT gene in plant development.

The important metabolic role of the OAT was clearly shown in an experiment where OAT-deficient plants failed to develop with arginine or ornitine as the sole nitrogen source (Funck et al., 2008). This result demonstrated that OAT is required for utilization of arginine and ornithine. The present study demonstrates high OAT promoter activity and elevated OAT transcript level during seed germination. These results are in agreement with available transcriptomic data (Winter D. et al., 2007). In arginine catabolism, OAT acts downstream of arginase (Funck et al., 2008). Arginine is regarded as a major nitrogen storage compound in seeds. Urease and arginase activities increase sharply during germination in A. thaliana (Zonia et al., 1995) and other plant species (Winter G. et al., 2015). Taken together, these data provide evidence for OAT involvement in nitrogen reorganization during seed germination together with other enzymes of arginine catabolism.

Our work also shows that the OAT gene promoter is active during inflorescence development. This observation is in accordance with recent findings showing that the OAT enzyme plays a role in flower development and seed setting in rice (Liu et al., 2018). It has been reported that rice plants with a mutated OAT gene (OsOAT mutants) have different abnormalities in inflorescence and seed development. The mutant phenotype of the OsOAT mutant could be rescued by application of urea (Liu et al., 2018). Authors assumed that OAT mediates arginase activity and plays a role in regulation of nitrogen reutilization, which is critical for developing tissues (Liu et al., 2018).

Taking into account the association between the OAT gene expression and proline accumulation (Roosens et al., 1998, 2002; Wu et al., 2003), it can be assumed that OAT enzyme activity may also play a role in control of proline level during inflorescence development. It has been reported that some proline metabolic enzymes can regulate a number of developmental processes including flowering time (Mattioli et al., 2008), pollen development (Mattioli et al., 2012; Biancucci et al., 2015a) and root growth (Biancucci et al., 2015b). Proline is known to be accumulated in reproductive organs of many plant species (Kavi Kishor et al., 2015). Ornithine to proline conversion is mediated by the plant oncogene RolD (Trovato et al., 2001), the overexpression of which stimulates flowering and affects inflorescence architecture in transgenic tobacco plants (Mauro et al., 1996). This study shows that the OAT promoter is active in inflorescences on different developmental stages (see Fig. 1), suggesting that the OAT enzyme can convert ornithine to proline directly or indirectly via arginine catabolism and glutamate production and might serve as a regulator of proline level during inflorescence development.

Tissue-specific activation of the OAT transcription in roots in response to auxin and cytokinin treatments, as well as the presence of the auxin-responsive element in the OAT promoter (see Suppl. Table 3) allow us to assume specific regulation of the OAT gene during root growth and development. Recent findings show the importance of nutrient and especially nitrogen signaling for root development and its interplay with hormone regulation. Thus, cytokinins negatively regulate uptake of nitrogen, but enhance nitrate distribution and translocation (Gu et al., 2018). Auxin level was shown to be elevated in roots of plants growing on a low-nitrogen medium, while in roots of plants growing on a medium with high nitrate concentration the auxin level was decreased. The reduction of auxin content correlated with the degree of inhibition of root growth and lateral root development (Kiba et al., 2011). The root growth regulation is associated with local reorganization of nitrogen metabolism (Kiba, Krapp, 2016). Thereby, OAT may play an important role in hormone-dependent fine-tuning of nitrogen metabolism during the process of root development.

## Conclusion

The data regarding the OAT transcription activation in a wide spectrum of experimental conditions, which was shown in our experiment and other studies, support the hypothesis that ornithine aminotransferase provides a link between different biochemical pathways of nitrogen conversion and contributes to the complicated signaling network regulating plant development.

## Conflict of interest

The authors declare no conflict of interest.
